# Inflammation-based score in pediatric adrenocortical carcinoma

**DOI:** 10.1530/ERC-24-0244

**Published:** 2025-03-28

**Authors:** Maria Riedmeier, Jan Idkowiak, Heidi Frey, Sonir R R Antonini, Gabriela Fernandes Luiz Canali, Carl Friedrich Classen, Nerea Domínguez-Pinilla, Martin Fassnacht, Steffen Fuchs, Christoph Härtel, Dominika Janús, Ronald de Krijger, Tezer Kutluk, Ngoc Lan Bui, Jagdish Prasad Meena, Mouna Mezoued, Jessica Munarin, Max M van Noesel, Nihal Özdemir Köse, Simon H Pearce, Thomas Perwein, Soraya Puglisi, Jaydira Del Rivero, Paul G Schlegel, Irene Schmid, Gerdi Tuli, Justyna Walenciak, Bilgehan Yalcin, Verena Wiegering

**Affiliations:** ^1^ University Hospital Würzburg, Department of Pediatrics, Division of Pediatric Hematology, Oncology and Stem Cell Transplantation, University of Wuerzburg, Wuerzburg, Germany; ^2^ Department of Metabolism and Systems Science, School of Medicine and Health, University of Birmingham, Birmingham, UK; ^3^ Department of Endocrinology and Diabetes, Birmingham Women’s and Children’s NHS Foundation Trust, Birmingham, UK; ^4^ Centre of Endocrinology, Diabetes and Metabolism, Birmingham Health Partners, University of Birmingham, Birmingham, UK; ^5^ Department of Pediatrics, Ribeirao Preto Medical School, University of Sao Paulo, Ribeirão Preto, Brazil; ^6^ Oncologia, Pequeno Principe Hospital, Curitiba, Paraná, Brazil; ^7^ Division of Pediatric Oncology, Hematology and Palliative Medicine Section, Department of Pediatrics and Adolescent Medicine, University Medicine Rostock, Rostock, Germany; ^8^ Pediatric Hematology and Oncology Unit, University Hospital 12 de Octubre, i+12 Research Institute, Madrid, Spain; ^9^ Department of Medicine, Division of Endocrinology and Diabetes, University Hospital, University of Wuerzburg, Wuerzburg, Germany; ^10^ Comprehensive Cancer Centre Mainfranken, University of Wuerzburg Medical Centre, Wuerzburg, Germany; ^11^ Department of Pediatric Oncology and Hematology, Charité – Universitätsmedizin Berlin, Berlin, Germany; ^12^ Berlin Institute of Health at Charité – Universitätsmedizin Berlin, Berlin, Germany; ^13^ German Cancer Consortium (DKTK), partner site Berlin, a partnership between DKFZ and Charité-Universitätsmedizin Berlin, Berlin, Germany; ^14^ Department of Pediatric and Adolescent Endocrinology, Jagiellonian University Medical College, University Children Hospital, Krakow, Poland; ^15^ Department of Pathology, University Medical Center Utrecht, Utrecht, The Netherlands; ^16^ Princess Maxima Center for Pediatric Oncology, Utrecht, The Netherlands; ^17^ Department of Pediatric Oncology, Hacettepe University Faculty of Medicine, Ankara, Turkey; ^18^ Pediatric Oncology Hematology Center, Vietnam National Children’s Hospital (VNCH), Hanoi, Vietnam; ^19^ Division of Pediatric Oncology, Department of Pediatrics, Mother & Child Block, All India Institute of Medical Sciences, New Delhi, India; ^20^ Department of Endocrinology and Metabolism, Bologhine Hospital, Algiers, Algeria; ^21^ Research Laboratory of Endocrinology and Metabolism (LEM1), Benyoucef Benkhedda University, Algiers, Algeria; ^22^ Department of Pediatric Endocrinology, Regina Margherita Children’s Hospital, Turin, Italy; ^23^ Department of Pediatrics, University of Turin, Turin, Italy; ^24^ Department of Endocrinology, Translational and Clinical Research Institute, Newcastle University, Newcastle upon Tyne, UK; ^25^ Division of Pediatric Hemato-Oncology, Department of Pediatrics and Adolescent Medicine, Medical University of Graz, Graz, Austria; ^26^ Internal Medicine, Department of Clinical and Biological Sciences, S. Luigi Gonzaga Hospital, University of Turin, Orbassano, Italy; ^27^ Developmental Therapeutics Branch, Center for Cancer Research, National Cancer Institute, National Institutes of Health, Bethesda, Maryland, USA; ^28^ Department of Pediatric Oncology and Hematology, Dr von Hauner Children’s Hospital, Ludwig-Maximilians-University Munich, Munich, Germany; ^29^ Department of Pediatrics, Oncology and Hematology, Medical University of Lodz, Lodz, Poland; ^30^ Mildred Scheel Early Career Center, University Hospital Wuerzburg, Wuerzburg, Germany

**Keywords:** inflammation-based score, prognostic factor, pediatric adrenocortical tumor, pediatric adrenocortical carcinoma

## Abstract

Inflammation-based scores have been demonstrated to be independent prognostic factors in predicting outcomes in adult adrenocortical carcinoma (ACC). We aimed to investigate the prognostic role of these scores in pediatric adrenocortical carcinoma (pACC) patients. An international multicenter analysis was conducted on a pediatric cohort from 21 ACC centers. Pretreatment inflammation-based scoring parameters, including neutrophil-to-lymphocyte ratio (NLR), derived neutrophil-to-lymphocyte ratio (dNLR), platelet-to-lymphocyte ratio (PLR), monocyte-to-lymphocyte ratio (MLR) and serum albumin, as well as clinical parameters, were analyzed. The primary endpoint was 10-year overall survival (OS). One hundred twenty-nine pediatric patients (50.4% females, mean age 87 months) across all tumor stages with a median follow-up of 36 months were included. 107/108 patients underwent primary surgery, and 62/106 received systemic treatment at the time of diagnosis. Of 102 patients, 27 died from disease. In the univariable analysis, NLR ≥5 (HR 8.0, 95% CI 3.4–19.1), MLR ≥0.28 (HR 4.2, 95% CI 1.7–10.4), PLR ≥190 (HR 4.5, 95% CI 2.0–10.4) and dNLR ≥1.44 (HR 5.9, 95% CI 2.3–15.5), as well as clinical parameters age ≥4 years (HR 5.5, 95% CI 1.9–15.8), tumor stage IV (HR 5.7, 95% CI 2.7–11.9) and incomplete resection status (HR 8.0, 95% CI 3.6–17.7) were significantly associated with reduced 10-year OS. After multivariable adjustment, only tumor stage IV (HR 336.7, 95% CI 5.8–19,518.1) and MLR ≥0.28 (HR 247.1, 95% CI = 3.1–19,907.5) were significantly associated with an unfavorable outcome. Inflammation-based scores tend to have prognostic value in pACC and could serve as prognostic tools after further validation in future studies with sufficient case numbers.

## Introduction

Pediatric adrenocortical carcinoma (pACC) is extremely rare, with an estimated yearly incidence of only 0.2–0.3 cases per million children (McAteer *et al.* 2013, Siegel *et al.* 2014). The tumor is strongly associated with TP53-related tumor syndromes, such as Li–Fraumeni syndrome, particularly in Southern Brazil due to the inherited TP53p.R337H germline mutation, which significantly increases tumor incidence in this region (Ribeiro *et al.* 2001, Pinto *et al.* 2004, Wasserman *et al.* 2015). It is known that patients with pACC differ clinically and histopathologically from their adult counterparts. Among other differences, almost all pediatric tumors produce androgens and/or cortisol, resulting in clinical presentations of virilization and/or Cushing syndrome (Michalkiewicz *et al.* 2004, Riedmeier *et al.* 2021). Surgical resection of the tumor is the fundamental therapeutic pillar for all tumor stages. Systemic chemotherapy and treatment with mitotane are used in advanced tumor stages (Redlich *et al.* 2012, Rodriguez-Galindo *et al.* 2021, Uttinger *et al.* 2022). Effective established personalized treatment options for advanced tumor stages do not currently exist. The prognosis for advanced tumor stages is unfavorable, and risk stratification remains challenging. Known prognostic factors include age, hormonal production, tumor size and stage, pathological parameters such as Ki-67 and resection status (Zambaiti *et al.* 2021, Riedmeier *et al.* 2024).

Chronic inflammation is involved in the development and progression of cancer (Coussens & Werb 2002, Mantovani *et al.* 2008). Peripheral differential blood-derived inflammation-based scores have been reported to predict outcomes in several different types of solid cancers, although the best cutoff values and thresholds vary among different cancer types and clinical patient characteristics (Mantovani *et al.* 2008, Templeton *et al.* 2014, Yodying *et al.* 2016, Dolan *et al.* 2017, Capone *et al.* 2018, Dupré & Malik 2018, Zhang *et al.* 2021, Mangone *et al.* 2023, Mangone *et al.* 2024). Inflammation-based scores, as shown in several studies, appear to be independent prognostic factors in predicting clinical outcomes in adult adrenocortical carcinoma (ACC) patients (Bagante *et al.* 2015, Mochizuki *et al.* 2017, Gaitanidis *et al.* 2019, Sisman *et al.* 2020, de Jong *et al.* 2021, Solak *et al.* 2021). Mangone *et al.* recently highlighted the role of these scoring parameters in predicting the response to systemic treatment and time to progression in adult patients with advanced ACC (Mangone *et al.* 2023) and demonstrated the relationship between inflammation-based scores and steroid production in these patients (Detomas *et al.* 2022, Favero *et al.* 2023, Mangone *et al.* 2024). To the best of our knowledge, no studies on inflammation-based scores in pediatric ACC patients have been published so far. Therefore, we conducted this study to analyze the role of inflammation-based scoring in predicting overall survival (OS) in pediatric ACC.

## Methods

International retrospective data of 129 pediatric patients with histopathologically proven pediatric adrenocortical carcinoma were collected from 21 international centers across 13 countries (5 × Germany, 2 × Brazil, 2 × Italy, 2 × Poland, 2 × UK, Turkey, USA, India, Algeria, Vietnam, Netherlands, Austria, Spain). We included patients of all tumor stages with and without systemic treatment for whom white blood cell differential (WBCD) counts at diagnosis were available. Notably, the systemic treatment regimen was heterogeneous due to the absence of internationally standardized guidelines for the treatment of pACC. Patients without follow-up data, as well as patients with known severe infections at the time of blood testing, were excluded. The primary endpoint of the study was OS – defined as the time from the first diagnosis to death or the last follow-up. Complete remission was characterized by the absence of disease at the last follow-up.

In the descriptive analysis of baseline characteristics in Table 1, patients without follow-up data were included. However, for the univariable and multivariable analyses of clinical, histopathological and inflammation-based scoring parameters predictive of OS, only patients with follow-up data were considered. In addition, the number of patients with available data for each parameter varied considerably, which accounts for the differences in patient numbers across the various analyses.

**Table 1:** Clinical characteristics of an international, multicenter cohort of 129 pediatric ACC patients.

Clinical characteristics^1^		Total	No systemic treatment	Systemic treatment	*P*
Number of patients		129^2^	44^2^	62^2^	
Sex f/m (% f)		65/64; 50.4	19; 43.2	35; 56.5	0.250^3^
Age at diagnosis (months) (median/range)		67/1–288	47/3–216	79/1–288	**0.024** * ^**,**^ **^4^**
Ten-year follow-up (months) (median/range)		36/2–120	45/9.97–120	32/2–120	0.029* ^**,**^ **^4^**
Ki-67 in % (median/range)		17.5/1–80	10/1–60	25/5–80	0.001** ^**,**^ **^4^**
Tumor stage (*n*; %)	I	27; 21.6	15; 34.9	4; 6.8	0.001** ^**,**^ **^5^**
	II	29; 23.2	16; 37.2	9; 15.3	
	III	32; 25.6	5; 11.6	22; 37.3	
	IV	37; 29.6	7; 16.3	24; 40.7	
	n.a.	4	1	3	
Hormonal secretion	Androgen	43; 43.4	16; 37.2	27; 48.2	0.472^5^
	Cortisol	16; 16.2	8; 18.6	8; 14.3	
	Mixed	29; 29.3	14; 32.6	15; 26.8	
	None	11; 11.1	5; 11.6	5; 10.7	
	n.a.	30	1	6	
Systemic treatment	Mitotane alone	12	0	12	
	Mitotane + chemotherapy	49	0	49	
	Chemotherapy alone	1	0	1	
	Radiotherapy	11	0	11	
Resection status (*n*; %)	R0	70; 65.4	33; 84.6	33; 56.0	0.018* ^**,**^ **^5^**
	R1	13; 12.1	0; 0	8; 13.6	
	R2	10; 9.3	3; 7.7	7; 12.0	
	Spillage	14; 13.1	3; 7.7	10; 16.9	
	Not resected	1	0	1	
Relapse (*n*; %)	Yes	35; 36.1	1; 2.8	34; 55.7	0.001** ^**,**^ **^4^**
	No	62; 63.9	35; 97.2	27; 44.3	
	n.a.	32	8	1	
Long-term outcome (*n*; %)	No evidence of disease	60; 58.8	34; 79	26; 44.1	0.001** ^**,**^ **^5^**
	AWD	12; 11.8	0; 0	12; 20.3	
	DOD	27; 26.5	6; 14	21; 35.6	
	LFU	3; 2.9	3; 7	0; 0	
	n.a.	27	1	3	
Origin (*n*; %)	Brazilian	19; 17.9	0; 0	19; 30.6	0.001** ^**,**^ **^3^**
	Non-Brazilian	87; 82.1	44; 100	43; 69.4	

^1^ Clinical characteristics including number of patients, sex, age at diagnosis, follow-up, Ki-67 status, tumor stage, resection stage, metastases, relapse, radiotherapy and outcome of the entire cohort as well as of the particular treatment subgroups (no systemic treatment, systemic treatment). Abbreviations: f, female; m, male; n.a., information not available; AWD, alive with disease; DOD, dead of disease; LFU, lost to follow-up.

^2^ Twenty-three of 129 patients were excluded from the treatment subgroup analysis due to the unavailability of data.

^3^ Continuity correction Yates test.

^4^ Kruskal–Wallis H test.

^5^ Chi-Square test were used.

* *P* < 0.05.

** *P* < 0.01.

Bold indicates statistical significance, *P* < 0.05.

Generally, WBCD counts for inflammation-based scores were collected before treatment start. Adapted to the previous literature (Mantovani *et al.* 2008, Templeton *et al.* 2014, Yodying *et al.* 2016, Dolan *et al.* 2017, Capone *et al.* 2018, Dupré & Malik 2018, Zhang *et al.* 2021, Mangone *et al.* 2023, Mangone *et al.* 2024), calculation of inflammation-based scores from WBCD counts was conducted as follows: neutrophil-to-lymphocyte ratio (NLR) as neutrophils divided by lymphocyte count, derived neutrophil-to-lymphocyte ratio (dNLR) as neutrophils divided by (leukocyte minus neutrophil count), platelet-to-lymphocyte ratio (PLR) as platelets divided by lymphocyte count, monocyte-to-lymphocyte ratio (MLR) as monocytes divided by lymphocyte count and albumin. Cutoff values for the WBDC-derived parameters NLR, PLR and albumin were used from the current literature on ACC (Bagante *et al.* 2015, Mochizuki *et al.* 2017, Grisanti *et al.* 2021, Zhang *et al.* 2021, Mangone *et al.* 2023). As cutoffs for MLR and dNLR were not yet established for ACC, the median values observed in the pediatric cohorts were used (1.44 for dNLR and 0.28 for MLR). The project was approved by the local ethics committee Würzburg (2023101601).

Statistical analyses were performed using RStudio to evaluate the findings of the study. The Kolmogorov–Smirnov test was used to determine the normality of the distribution of the parameters. Descriptive statistical methods (median, range) were applied, and the Kruskal–Wallis test was used for comparisons between groups with non-normally distributed parameters, followed by the Mann–Whitney U test to determine the group causing the difference. The Mann–Whitney U test was also used for comparisons of non-normally distributed numerical variables between the two groups. Chi-square test and Yates’ continuity correction test were used for the comparison of qualitative data. Survival curves were constructed by the Kaplan–Meier method, and the results were compared by univariate and multivariate Cox regression analysis. Results are presented as hazard ratio (HR) with 95% confidence intervals (95% CI). Statistical significance was conventionally set at *P* < 0.05.

## Results

### General patient characteristics

This study included 129 cases with a median 10-year follow-up of patients of 36 months (range: 2–120 months). Sixty-five patients (50.4%) were female. The majority, 82.1%, were non-Brazilian patients and the median age at diagnosis was 67 months (range: 1–288 months), with a mean age of 87.1 months. The median proliferation index Ki-67 was 17.5% (range: 1–80%). The distribution of patients across tumor stages I–IV (Sandrini *et al.* 1997, Fassnacht *et al.* 2009) was approximately equal, with a slight predominance of advanced stages. Almost all patients (88/99, 88.8%) had hormone-secreting tumors, with the majority predominantly secreting androgens (*n* = 43, 43.4%). One hundred seven of 108 (99.1%) patients underwent primary surgery. Most of these patients (*n* = 70/107, 65.4%) received R0 resection, while tumor spillage occurred in 14 patients (13.1%). Thirty-five of 97 patients with available information (36.1%) experienced relapse in the long-term follow-up. Regarding outcomes, the majority of patients (*n* = 60/102, 58.8%) had no evidence of disease at the last follow-up. However, 12/102 patients (11.8%) were alive with disease, 27/102 patients (26.5%) died of disease and 3/102 patients (2.9%) were lost to follow-up (for details see Table 1).

**Figure 1:**
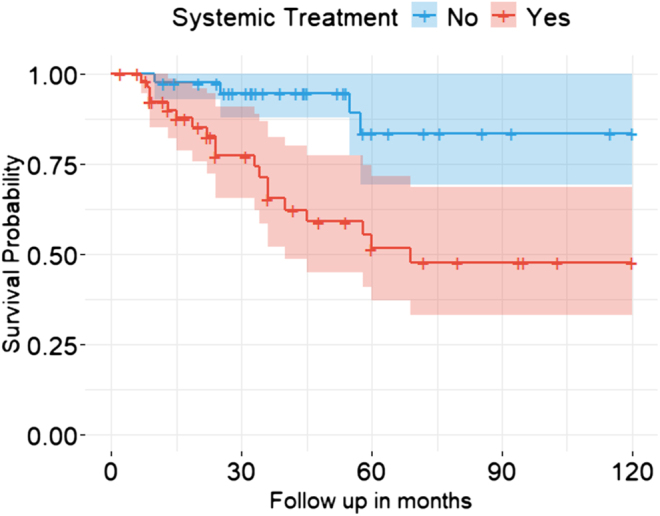
Ten-year OS of pACC patients by treatment. Kaplan–Meier curves depicting OS from diagnosis (follow-up in months) for pediatric patients by treatment groups (blue: patients without systemic treatment, red: patients with systemic treatment), comparisons between survival curves were analyzed with Cox regression.

Regarding the treatment regimen, 62/106 patients (58.5%) received systemic treatment at the time of primary diagnosis, including chemotherapy and/or mitotane therapy and/or radiotherapy, while 44/106 patients (41.5%) did not receive any systemic treatment. Detailed treatment information was unavailable for 23 cases. Significant differences were observed between the two groups (see Table 1). Patients who received systemic treatment were significantly older at diagnosis compared to those who did not receive systemic therapy (*P* = 0.024). The group receiving systemic treatment exhibited a significantly higher rate of advanced tumor stages, incomplete resection status, elevated Ki-67 levels, greater frequency of relapse, fewer cases of complete remission and a higher incidence of metastasized tumors compared to the group not treated with systemic therapy (all *P* < 0.05). OS differed significantly between the two groups, with the survival rate of patients not receiving systemic treatment being significantly higher than those receiving systemic therapy (HR 3.9, CI 95% 1.4–10.4, *P* < 0.01) (see Fig. 1).

### Inflammation-based score as predictor of OS

**Figure 2:**
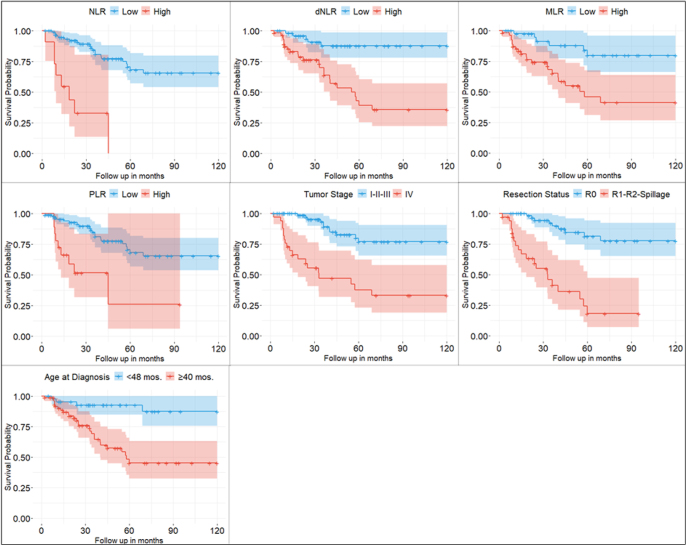
Ten-year OS of pACC patients by NLR, dNLR, MLR, PLR, tumor stage, resection status and age. Kaplan–Meier curves showing OS from diagnosis (follow-up in months) for pediatric patients regardless of treatment (*n* = 129). NLR = neutrophil-to-lymphocyte ratio (high risk≥ 5), dNLR = derived neutrophil-to-lymphocyte ratio (high risk≥ 1.44), MLR = monocyte-to-lymphocyte ratio (high risk≥ 0.28), PLR = platelet-to-lymphocyte ratio (high risk ≥190), tumor stage, resection status (high risk > R0), and age at diagnosis (high risk ≥4 years). Comparisons between survival curves were analyzed with Cox regression.

Survival analyses were conducted for the entire patient cohort as well as for the particular treatment subgroups (with and without systemic treatment), incorporating clinical, histopathological and inflammation-based factors. In the univariable analysis of all patients, irrespective of treatment, age at diagnosis ≥4 years, advanced tumor stage (stage IV), incomplete resection status, NLR ≥5, dNLR ≥1.44, PLR ≥190 and MLR ≥0.28 were significantly associated with inferior 10-year OS (see Table 2 and Fig. 2). In the multivariable analysis, only tumor stage IV (HR = 336.7, 95% CI = 5.8–19,518.1) and MLR ≥0.28 (HR = 247.1, 95% CI = 3.1–19,907.5) remained significant prognostic factors of OS. No significant association was found between OS and Ki-67, cortisol secretion or serum albumin levels (see Table 2).

**Table 2:** Univariable and multivariable analysis of clinical, histopathological and inflammation-based scoring factors predictive of 10-year overall survival of all pACC patients.

Prognostic factors^1^	N_1_/N_2_/N_3_/N_4_/*n*_1_/*n*_2_/*n*_3_^2^	Univariable			Multivariable		
		HR	95% CI	*P*	HR	95% CI	*P*
Age at diagnosis ≥48 months	78/44/10/8/27/4/7	5.5	1.9–15.8	0.001**	2.8	0.02–372.5	0.682
Tumor stage IV	36/84/6/12/19/11/5	5.7	2.7–11.9	0.001**	**336.7**	**5.8**–**19,518.1**	0.005**
Resection status = 1,2, spillage	35/68/10/8/20/9/6	8.0	3.6–17.7	0.001**	1.7	0.3–0.7	0.498
Ki-67% ≥20	40/50/2/4/12/9/2	2.2	0.9–5.2	0.079	0.01	0–0.3	0.052
NLR ≥5	11/100/2/14/8/21/2	8.0	3.4–19.1	0.001**	2.9	0.2–35.8	0.395
dNLR ≥1.44	59/52/8/8/24/5/6	5.9	2.3–15.5	0.001**	0.3	0–51.3	0.654
PLR ≥190	19/92/3/13/9/20/2	4.6	2.0–10.4	0.001**	15.2	0.4–537.2	0.135
MLR ≥0.28	57/45/7/7/22/6/6	4.2	1.7–10.4	0.002**	**247.1**	**3.1**–**19,907.5**	**0.014** *
Albumin ≤39	31/61/4/6/9/14/2	1.6	0.7–3.6	0.300	0.4	0.1–2.5	0.327
Cortisol secretion	17/99/1/17/23/1	1.7	0.7–3.9	0.220	2.5	0.2–30.7	0.469

^1^ Prognostic factors including age at diagnosis, tumor stage, resection status, Ki-67 and inflammation-based factors (NLR, dNLR, PLR, MLR and albumin) including the entire patient cohort (129 patients).

^2^ Abbreviations: N_1_: number of patients with the relevant criterion and available follow-up data; N_2_: number of patients out of the relevant criterion and with available follow-up data; N_3_: number of Brazilian patients with the relevant criterion and available follow-up data; N_4_: number of patients out of the relevant criterion and with available follow-up data; *n*_1_: frequency of death in the relevant criterion; *n*_2_: frequency of death out of the relevant criterion; *n*_3_: frequency of death in Brazilian patients in the relevant criterion; HR: hazard ratio; CI: confidence interval.

* *P* < 0.05.

** *P* < 0.01.

Bold indicates statistical significance, *P* < 0.05.

Analyzing the particular treatment groups, age at diagnosis, resection status, NLR, dNLR and MLR successfully predicted 10-year OS in the univariable analysis for the pediatric cohort with systemic treatment. Tumor stage, Ki-67, PLR and albumin level were not significantly associated with 10-year OS (see Table 3). Tumor stage, Ki-67 and MLR remained significant in the multivariable Cox regression analysis (see Table 3). In this cohort without systemic treatment, none of the factors significantly predicted survival (*P* > 0.05) (data not shown).

**Table 3:** Univariable analysis of clinical, histopathological and inflammation-based scoring factors predictive of 10-year overall survival of pACC patients with systemic treatment.

Prognostic factors^1^	N_1_/N_2_/N_3_/N_4_/*n*_1_/*n*_2_/*n*_3_^2^	Univariable			Multivariable		
		HR	95% CI	*P*	HR	95% CI	*P*
Age at diagnosis ≥48 months	39/17/10/8/16/3/7	4.2	1.2–14.5	0.025*	2.6	0–375.3	0.706
Tumor stage IV	23/31/6/12/10/8/5	1.9	0.7–4.8	0.186	**186.4**	**5.2**–**6,720.8**	0.004**
Resection status = 1,2, spillage	23/31/10/8/10/8/6	2.6	1.0–6.7	0.045*	1.7	0.3–8.0	0.525
Ki-67% ≥20	21/10/2/4/6/4/2	0.7	0.2–2.6	0.608	**0.2**	**0**–**0.4**	0.010*
NLR ≥5	5/46/2/14/4/14/2	5.1	1.6–16.4	0.006**	3.5	0.3–44.5	0.329
dNLR ≥1.44	30/21/8/8/15/3/6	5.4	1.5–18.7	0.008**	0.7	0–78.9	0.864
PLR ≥190	10/41/3/13/5/13/2	2.7	1.0–7.8	0.062	8.7	0.4–212.4	0.184
MLR ≥0.28	28/30/7/7/14/3/6	4.2	1.2–14.9	0.024*	**100.5**	**3.1**–**3,234.5**	0.009**
Albumin ≤39	17/22/4/6/4/7/2	0.8	0.2–2.8	0.736	0.5	0.1–2.6	0.404

^1^ Prognostic factors including age at diagnosis, tumor stage, resection status, Ki-67 and inflammation-based factors (NLR, dNLR, PLR, MLR and albumin) of pACC patients with systemic treatment (62 patients).

^2^ Abbreviations: N_1_: number of patients with the relevant criterion and available follow-up data; N_2_: number of patients out of the relevant criterion and with available follow-up data; N_3_: number of Brazilian patients with the relevant criterion and available follow-up data; N_4_: number of patients out of the relevant criterion and with available follow-up data; *n*_1_: frequency of death in the relevant criterion; *n*_2_: frequency of death out of the relevant criterion; *n*_3_: frequency of death in Brazilian patients in the relevant criterion; HR: hazard ratio; CI: confidence interval.

* *P* < 0.05.

** *P* < 0.01.

Bold indicates statistical significance, *P* < 0.05.

Focusing on NLR as a component of the inflammation-based score, NLR levels were significantly higher in patients with cortisol-producing tumors compared to those with non-cortisol-producing tumors, as well as in high-malignancy tumors compared to low-malignancy ones (see Table 4).

**Table 4:** Association of NLR levels with tumor stage and hormonal secretion in pACC patients.

		NLR*		
		Mean ± SD	Median/range	*P*-value
Tumor stage	I–II (low malignancy)	1.91 ± 1.45	1.61/7.21	0.004**
	III–IV (high malignancy)	2.82 ± 2.07	2.28/10.80	
Hormonal secretion	No hormones/only androgen	1.88 ± 1.39	1.76/6.84	0.001**
	Cortisol/mixed	5.09 ± 13.05	2.8/98.01	

* Mann–Whitney U test; SD, standard deviation.

** *P* < 0.01.

**Figure 3:**
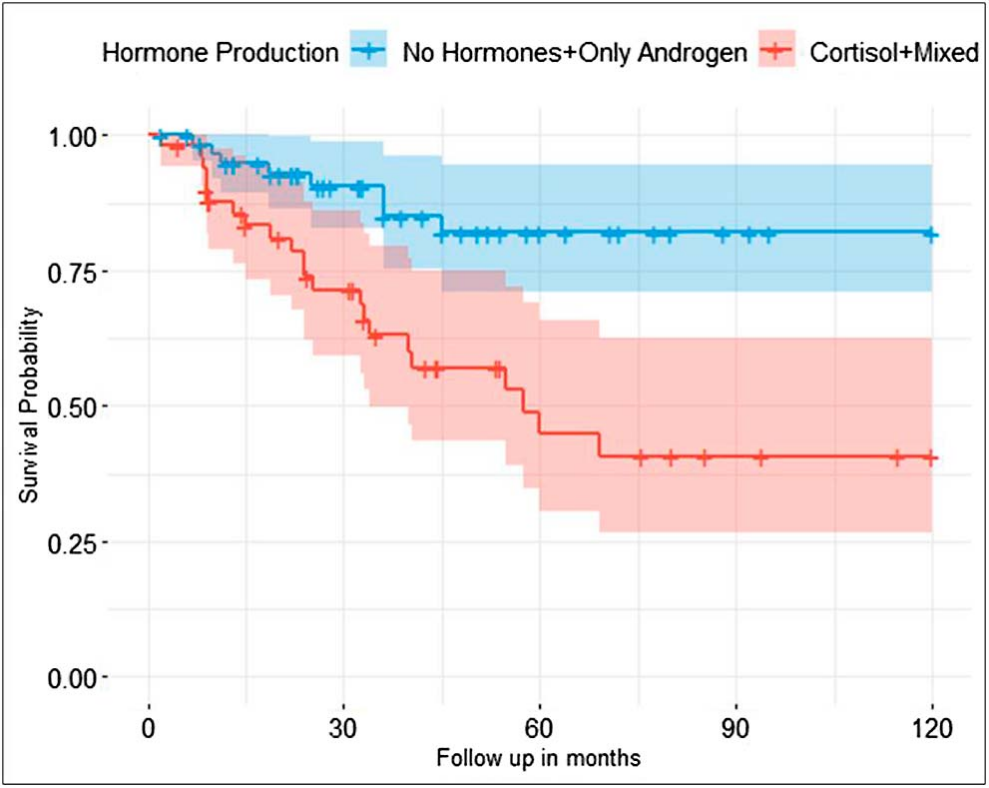
Ten-year OS of pACC patients by hormone production. Kaplan–Meier curves depicting OS from diagnosis (follow-up in months) for pediatric patients by hormone groups (blue: no hormones/only androgen-producing tumors, red: cortisol-producing/mixed hormones-producing tumors). Comparisons between survival curves were analyzed with Cox regression.

To investigate the prognostic value of cortisol secretion, we also performed survival analysis for patient cohorts grouped by hormone production (group 1: cortisol and mixed hormone production (*n* = 52), group 2: no hormone and only androgen production (*n* = 64)). OS was significantly worse in the cortisol/mixed hormone production group compared to the group without cortisol production (*P* < 0.05) (see Fig. 3). In the univariable analysis of pACC patients with non-hormone-secreting or only androgen-secreting tumors, tumor stage, resection status, NLR, dNLR and PLR were significantly associated with 10-year OS (see Supplement Table 1 (see section on Supplementary materials given at the end of the article)). In contrast, in the univariable analysis of pACC patients with cortisol-producing tumors, these same factors, along with age at diagnosis and Ki-67, had a significant impact on OS (see Supplement Table 2). However, due to the limited number of patients, it is not possible to draw definitive conclusions about the influence of cortisol production on the prognostic value of inflammation-based scores.

## Discussion

Risk stratification of pACC patients remains challenging despite the evaluation of several clinical and histopathological parameters as prognostically relevant (Riedmeier *et al.* 2022, 2024). Identification of further prognostic factors is necessary to enable improved risk stratification of pACC patients, especially in advanced tumor stages. To the best of our knowledge, this is the first study investigating the prognostic role of inflammation-based scores in predicting OS in pACC.

Chronic inflammation is connected to the tumor microenvironment and plays a role in tumor development and progression (de Visser & Coussens 2006, Singh *et al.* 2019). As a result, the pathophysiological interactions between the inflammatory tumor stroma and cancer cells are increasingly studied as prognostic biomarkers (Coussens & Werb 2002, Mantovani *et al.* 2008). The influence of cancer-related inflammation on tumor behavior is integrated into inflammation-based scores, which have been demonstrated to predict clinical outcomes in cancer (Mantovani *et al.* 2008, Templeton *et al.* 2014, Yodying *et al.* 2016, Dolan *et al.* 2017, Capone *et al.* 2018, Dupré & Malik 2018, Zhang *et al.* 2021, Mangone *et al.* 2023, 2024).

In studies focused on ACC, NLR has been suggested to independently play a prognostic role. NLR was higher in carcinoma than adenoma (Mochizuki *et al.* 2017, Gaitanidis *et al.* 2019, Sisman *et al.* 2020, Detomas *et al.* 2022), associated with larger tumors (Bagante *et al.* 2015), and predicted shorter OS, shorter progression-free survival, and poorer treatment response in adult patients with advanced ACC (Mangone *et al.* 2023). In addition, NLR predicted progression-free survival in patients with metastatic ACC treated with gemcitabine plus capecitabine (Mantovani *et al.* 2008). Our current study involving pediatric patients aligns with previous research on adult ACC patients. We showed that NLR tended to be a dependable predictor of OS in the univariable analysis including all patients. Similar to NLR, dNLR with a cutoff of 1.44 also correlated with worse patient outcomes in the univariable analysis, although it had a minimally less robust prognostic value, which is in concordance with previous studies about solid tumors even though cutoff values were different (Capone *et al.* 2018, Mangone *et al.* 2023).

Regarding PLR, our findings are consistent with previous studies (Bagante *et al.* 2015, de Jong *et al.* 2021, Solak *et al.* 2021), as this parameter also tended to impact OS in pACC patients. In contrast to the data of Mangone *et al.* (2023), we demonstrated the role of MLR in predicting OS in pediatric patients in the multivariable analysis. Regarding serum albumin, Zhang *et al.* showed that serum albumin has prognostic value in adult ACC patients after primary tumor resection (Mangone *et al.* 2023). We could not show prognostic relevance in pACC patients with cortisol-producing tumors in the univariable analysis. At this point, due to low data availability of the variable we cannot draw a definitive conclusion about the impact of this prognostic factor.

However, when analyzing specific treatment groups, statistical significance of these factors was observed only within the cohort receiving systemic treatment, likely due to the very limited number of patients in the group not treated systemically with available WBDC-derived parameters. An additional consideration could be that tumors with lower malignancy are associated with less inflammation, making the prognostic effect of the inflammation score less valid in earlier tumor stages.

Regarding clinical parameters, our data once again confirmed the prognostic role of age, tumor stage and resection status. Only Ki-67 failed to be significant, which might be due to the limited number of cases available for Ki-67 analysis.

Recent studies on the role of hormonal secretion in adult ACC patients have shown that cortisol excess impacts inflammation-based scores by causing hematological changes such as leukocytosis and neutrophilia (Detomas *et al.* 2022, Mangone *et al.* 2024). However, Mangone *et al.* recently demonstrated that a higher inflammation score reflects malignancy regardless of the hormonal production of the tumor (Mangone *et al.* 2024). As almost all pediatric ACCs are hormone-producing, even with a lower percentage of cortisol-producing tumors (Michalkiewicz *et al.* 2004, Riedmeier *et al.* 2021), we aimed to investigate the impact of cortisol production by the tumors in the pediatric cohort. To do this, we analyzed the hormone-producing cohorts separately. The study results indicated that patients with cortisol-producing tumors had significantly inferior OS compared to those with androgen-producing tumors or non-hormone-producing tumors. Our results align with the hypothesis of the relation of inflammation-based scores to cortisol production proposed by Mangone *et al.* (2024), as the NLR level was significantly higher in pediatric patients with cortisol-producing tumors. However, due to the limited number of patients and the design of the study, it is not possible to draw definitive conclusions about the influence of cortisol production on the prognostic value of inflammation-based scores. Our study also supports the hypothesis of Mangone *et al.* that inflammation scores reflect malignancy irrespective of hormone production (Mangone *et al.* 2024), as NLR levels were significantly higher in high-malignancy tumors compared to low-malignancy ones.

The current study has several limitations, as it is a retrospective analysis with a limited number of cases due to the extreme rarity of ACC in childhood. Therefore, the reliability of the results, at least for the particular sub-cohorts, was constrained. Due to the heterogeneity of the international cohort, systemic treatment regimens differed among the centers, as no international guidelines for the treatment of pACC currently exist. In addition, complete data on patient characteristics and inflammation scores were not available for all patients, leading to a highly variable number of patients across the different analyses. In particular, for parameters with low data availability, the analysis is less robust.

Further studies involving larger cohorts with standardized treatment regimens are necessary to validate our findings and to reach significant results in the multivariable analysis. Nevertheless, we were able to demonstrate for the first time that inflammation-based scores tend to have prognostic value in pediatric patients with ACC. Due to its ease of assessment and collection in clinical settings, the inflammation-based score could serve as a prognostic tool in the future, representing a potential advancement in the risk stratification of patients with pACC.

## Supplementary materials



## Declaration of interest

The authors declare that there is no conflict of interest that could be perceived as prejudicing the impartiality of the work reported.

## Funding

This work was supported by a research grant ‘Interdisziplinäres Zentrum für klinische Forschunghttps://doi.org/10.13039/501100009379 (IZKF)’ training grant awarded to MR (project number: Z-02CSP/23), the ‘Mildred Scheel program’ awarded to VW (Project 70113303-6), by the ‘Deutsche Forschungsgemeinschafthttps://doi.org/10.13039/501100001659’ (DFG) German Research Foundation (Project 314061271-TRR 205) to MF, and the National Institute of Health and Care Research, UK (Clinical Lecturership to JI). This work was also supported by a research grant from the Tour of Hope Foundation. The funders had no role in study design, data collection and analysis, decision to publish or preparation of the manuscript.
