# Supplementary material for: Inflammation-based score in pediatric adrenocortical carcinoma
Source: Endocr Relat Cancer. 2025 Mar 28;32(5):e240244. doi: 10.1530/ERC-24-0244 (PMC12002726; doi:10.1530/ERC-24-0244)
Supplement: Supplementary file 1 [file supplementary_materials.pdf]

Supplement Table 1: Univariable analysis of clinical, histopathological, and inflammation-based scoring factors predictive of 10-year overall survival of pACC patients with no hormone/ only androgen secretion.

**Prognostic factors**[1]		**Univariable**			**Multivariable**		
	**$N_1/N_2/N_3/N_4/n_1/n_2/n_3$**	**HR**	**95% CI**	**p**	**HR**	**95% CI**	**p**
Age at diagnosis ≥ 48 months	37/27/6/7/6/2/3	2.721	0.547 – 13.543	0.222	0.144	0-4.599E+182	0.993
Tumor stage IV	9/54/2/11/3/5/1	5.064	1.206 – 21.265	**0.027***	15959598.34	3.042E+97	0.876
Resection status= 1,2, spillage	17/33/6/7/2/5/2	7.558	1.458 – 39.181	**0.016***	2972.56	1.508E+65	0.912
Ki-67 % ≥ 20	21/27/2/3/3/5/2	1.103	0.262 – 4.643	0.894	0	1.556E+83	0.878
NLR ≥ 5	2/55/1/11/2/6/1	8.626	1.736 – 42.857	**0.008****	0	1.586E+81	0.881
dNLR ≥ 1.44	24/33/6/6/6/2/4	5.278	1.060 – 26.281	**0.042***	3755.446	1.398E+153	0.963
PLR ≥ 190	8/49/2/10/3/5/1	7.159	1.599 – 32.045	**0.010***	8478.751	3.513E+228	0.902
MLR ≥ 0.28	20/32/4/6/5/2/3	4.770	0.918 – 24.777	0.063	4.749	1.368E+228	0.995
Albumin ≤ 39	16/32/2/5/2/3/0	1.378	0.230 – 8.253	0.725	0	8.596E+80	0.877

[1]Prognostic factors including tumor stage, resection status, Ki67 and inflammation-based factors (NLR, dNLR, PLR, MLR, Albumin) of pACC patients with no hormone/ only androgen secretion (64 patients)

[2]Abbreviations: N_1: the number of patients with the relevant criterion and available follow-up data; N_2: the number of patients out of relevant criterion and with available follow-up data; N_3: number of Brazilian patients with the relevant criterion and available follow-up data; N_4: the number of patients out of relevant criterion and with available follow-up data; n_1: frequency of death in relevant criterion; n_2: frequency of death out of relevant criterion; n_3: frequency of death in Brazilian patients in relevant criterion; HR: Hazard Ratio; CI: Confidence Interval; **$p<0.01$; *$p<0.05$

Supplement Table 2: Univariable analysis of clinical, histopathological, and inflammation-based scoring factors predictive of 10-year overall survival of pACC patients with cortisol/ cortisol and androgen secretion.

**Prognostic factors**[1]		**Univariable**			**Multivariable**		
	**$N_1/N_2/N_3/N_4/n_1/n_2/n_3$**	**HR**	**95% CI**	**p**	**HR**	**95% CI**	**p**
Age at diagnosis ≥ 48 months	35/17/4/1/20/2/4	0.696	1.793 – 33.038	**0.006******	3448.98	0-7.413E+173	0.968
Tumor stage IV	24/27/4/1/16/5/4	4.647	1.694 – 12.746	**0.003****	425915.11	0-5.640E+62	0.847
Resection status= 1,2, spillage	17/33/4/1/15/6/4	10.473	3.735 – 29.367	**0.001****	0.355	0.025-5.054	0.444
Ki67 % ≥ 20	16/21/0/1/8/4/0	3.549	1.041 – 12.101	**0.043***	0	0-8.289E+51	0.859
NLR ≥ 5	9/40/1/3/6/14/1	5.524	1.931 – 15.805	**0.001****	956116389.7	0-4.167E+171	0.914
dNLR ≥ 1.44	32/17/2/2/17/3/2	4.311	1.260 – 14.754	**0.020***	192.541	1.355E+92	0.960
PLR ≥ 190	10/39/1/3/6/14/1	2.887	1.090 – 7.646	**0.033***	-	-	-
MLR ≥ 0.28	34/11/3/1/16/4/3	2.120	0.702 – 6.405	0.183	880559945.0	0-1.277E+79	0.803
Albumin ≤ 39	13/27/2/1/7/11/2	2.180	0.836 – 5.686	0.111	0	1.502E+151	0.958

[1]Prognostic factors including tumor stage, resection status, Ki67 and inflammation-based factors (NLR, dNLR, PLR, MLR, Albumin) of pACC patients with cortisol/ cortisol and androgen secretion (52 patients)

[2]Abbreviations: N_1: the number of patients with the relevant criterion and available follow-up data; n_1: frequency of death in relative criterion; n_2: frequency of death out of relative criterion; n_3: frequency of death in Brazilian patients in relative criterion; HR: Hazard Ratio; CI: Confidence Interval; **p<0.05,* **p<0.01
